# Vaccination in the Light of the Royal British Commission

**Published:** 1897-03

**Authors:** M. R. Leverson

**Affiliations:** Fort Hamilton, L. I.


### VACCINATION IN THE LIGHT OF THE ROYAL BRITISH COMISSION.

M. R. Leverson, M. D., Fort Hamilton, L. I.

(Continued from page 9.)

No one who has not waded through a mass of testimony, some of it irrelevant, much of it ambiguous, and much more that is contradictory, can realize the difficulty of extracting that which deserves notice from the rest. In studying the enormous volume of testimony given before the Royal (British) Commission of 1889, parts of it which may not at first seem to deserve notice, in the light of evidence given at a future date are seen to be important. I trust the readers of The Homœopathic Physician will accept this as an apology for sometimes retracing my steps to take up the testimony of persons already passed under review.

In 1857 and again in 1871 Sir John Simon said: "On the conclusion of this artificial disorder, neither renewed vaccination, nor inoculation with small-pox, nor the closest contact and cohabitation with small-pox patients will occasion him to betray any remnant of susceptibility to infection;" but in 1889 he says:

Q. 174. "If I were now writing the passage I should probably not go farther than to express my belief that the person might, without fear, expose himself to any risk of infection." And again (Q. 175) "My meaning was only as to the *immediate time.* I have no fresh knowledge what immediate vaccination would do, nor as to immediate inoculation with small-pox."

(Q. 177) He believes a vaccinated person is practically in the position of one who has had the small-pox, and (Q. 179) he does not know how soon after primary vaccination it is possible to re-vaccinate successfully, but (180–181) believes that ninety per cent. of the re-vaccinations of recruits entering the army are successful.

Some points of Dr. Ogle's testimony, to which I omitted to refer, ought, I think, to be presented to the readers of THE HOMŒOPATHIC PHSICIAN.

That gentleman states (Q. 346) that in 1871 the deaths from small-pox reached practically as high a level as in 1838, viz., 1,015 per million living.

It is to be remembered that this small-pox mortality followed in the teeth of many years of compulsory vaccination, wherein the vaccinations amounted to ninety six per cent. of the births and over ninety per cent. of the entire population of Great Britain were vaccinated. In 597 Dr. Ogle gives the opinion that vaccination is not so great a protection against small-pox as a previous attack of small-pox itself would be.

Then, surely, *compulsory inoculation* must, logically, be preferable to compulsory vaccination! M. Picton (626–635) draws from him the admission that small-pox has diminished *more than other diseases* in the "healthy" districts, and though Dr. Ogle discriminates between what the Registrar-General terms "healthy districts" and those in which sanitary improvements have been carried out (632–635), he admits (637–8) that the prevalence of small-pox in unsanitary districts, such as Liverpool, "certainly was connected with unsanitary conditions."

In Q. 509–515 Dr. Ogle says that the death-rate of adults from small-pox above the age of forty-five has increased, while the decline from fever has been common to all ages, but soon admits "that the decline (in fever) was greater among children than it was at other ages."

Before leaving the testimony of Mr. Richard Thorne Thorne, M. B., who proved himself to be the great "know-nothing" of the local Government Board, there are a few points deserving further notice.

He stated (Q. 820) that in pre-vaccination days from 80 to 90 per cent. of small-pox deaths occurred in children under five years of age. In the Registrar-General's returns for 1881–87, there were 3,099 deaths from small-pox of persons *returned as unvaccinated*, but of these only 1,210, or 39 per cent., were under five years of age (Q. 890). These facts do not fit in with Mr. Thorne's theories, and he is unable to explain them. The truth is they indicate what has been elsewhere proved, that the statistics of vaccinated or unvaccinated are wholly unreliable.

At Q. 901 Dr. Collins introduced a passage from Miss Florence Nightingale's *Notes on Nursing*, which is like an oasis of common sense in the desert of worthless statistics and ridiculous verbiage wherewith the medical vaccinists "darken counsel with words without wisdom." It is as follows: "I have seen with my eyes and smelt with my nose small-pox grow up in first specimens, either in close rooms or in overcrowded wards, where it could not by any possibility have been caught, but must have begun. Nay more, I have seen diseases begin, grow up, and pass into one another. I have seen, for instance, with a little overcrowding, continued fever grow up, and with a little more typhoid fever, and with a little more, typhus; and all in the same ward or hut."

Mr. Thorne, in Q. 744, had spoken of the effectiveness of vaccination being in part dependent upon the number of scars, but in answer to Q. 912–3, says he does not regard cow-pox as a local disease, but as affecting the whole system; he was unable to answer whether or not the cow-pox disease would be made more severe or acute by the same amount of matter being distributed over four incisions than it would be by that matter being put into one (Q. 914), and in Q. 915 he makes the startling confession that "We really know absolutely nothing as to the effect of the specific poisons upon the blood!"

At Q. 918 he says: "We attach the very greatest importance to the quality of the lymph," but admits (921) that even under the microscope good lymph is not distinguishable from bad; and we shall hereafter see that all the vaccinists admit that there is no way of telling "good vaccine" from bad *save by its effects.* Finally (1011–13), Dr. Thorne concedes that *isolation* has been effectual in preventing the spread of the disease, although in Q. 927 he says: "I believe that apart from that one influence of crowding together either on area or within dwellings, the influence of sanitary circumstances is so insignificant that it is hardly worth naming in connection with the subject that is now before the Commission."

In answer to Q. 495, and again in Q. 516, Dr. Ogle considered that the death-rate from symotic diseases or from typhoid should be compared with that from small-pox to determine whether the decline in either case was owing to sanitation or to vaccination, but, according to Dr. Thorne (Q. 875), such a comparison is utterly valueless for that purpose.

The next witness was the Surgeon-General in the service of the government of India, Dr. John Pinkerton, who had served in the vaccination department for thirteen years, during six of which he was Superintendent of Vaccination in India.

Dr. Pinkerton stated (Q. 1,229) that when he went to Scinde in 1863 "nearly the whole population were marked with small-pox, young and old; except the very young, the youths and old people were all marked." He also said (Q. 1,230) that blindness of one eye was so frequent a sequela of small-pox that it had given birth to a special word in the Scindi language for it, viz.: *Kanu.*

In reply to Q. 1,231 he said that vaccination was begun by the Bombay government in 1848, but was not pushed at all until 1857, because the bulk of the population were Mussulmen *who habitually practiced inoculation.* Vaccination was gradually introduced from 1848, and after eight or nine years was *pushed* by Dr. Martin. Dr. Pinkerton succeeded Dr. Martin in 1863, and under his régime the number of vaccinators was greatly increased, so that it covered the whole area. He went back to Scinde in 1882 as Deputy Surgeon-General, and remained there till 1888, and he told the Commission that he then observed that "the younger population was quite unmarked by small-pox."

It is surprising how the grossest absurdities will be greedily embraced, in perfect good faith too, although their absurdity has been over and over again exposed, when they happen to fit in with preconceived fancies, especially when the bias of personal interest is enlisted in their support.

The universally received theory that one attack of small-pox protects from a subsequent attack (a theory which never had the smallest basis in facts), ensured ready reception to the assumed prophylaxy of cow-pox when Jenner cunningly introduced to the profession, what to most of them was a new disease, styling it "small-pox of the cow" as though that were the name by which it had been known.

From that date the fable of pock-marked faces has been frequently repeated at various successive dates—such repetition being sufficient to establish its absurdity. The absence of pock-marked faces in the rising generation while they were "obvious everywhere" a few years previously was insisted on by the National Vaccine establishment in 1822 and 1825 as proof of the efficacy of vaccination. So, then, in 1822 and 1825 it was exceedingly rare to see a young person bearing the marks of small-pox; yet in 1837, the rarity of such faces as compared with what occurred a few years previous is again referred to for the same purpose, and from 1837 down to this day the same alleged fact is again and again repeatedly asserted, and "twenty years ago" is the date assigned when nearly every face one met bore upon it the scars of the terrible scourge.

As these assertions are mutually destructive it may be left to the vaccinists to explain them. In Dr. Pinkerton's statement we find also a cruel blow at the alleged anto-protection of small-pox. For "the bulk of the population was Mussulmen who habitually practiced inoculation for small-pox on the wrist," yet "nearly the whole population were marked with the small-pox."

In this connection it may be of use to refer to a case mentioned in *Le Médecin*, the official organ of the Belgian Medical School, of the date of January 17th, 1897.

A prisoner, No. 46, in the prison of Charleroi, thirty years old. 1st. Was vaccinated between two and three years of age with pronounced success. Four marks magnificently foveated. 2d. Scars on the face of an attack of small-pox at the age of eight years. 3d. Fresh scars also on the face of a second attack of confluent small-pox at the age of fifteen.

Dr. Pinkerton narrates a marvelous tale (Q. 1,237–8) of the escape of the vaccinated Mussulmen children of a village in

Hyderabad, while every one of the unvaccinated Hindu children took it. Experience of similar stories related as occurring in places rather more accessible to investigation than an *unnamed* village in Hyderabad, compels us to say that we don't believe it. Dr. Pinkerton further says that there were only fifteen deaths from small-pox in the period 1865–68 in the Bombay native army, amounting to about 35,000 men and under 40,000, putting it at 37,500, gives 400 deaths per million, or an annual average of 40 per million living.

These troops were of course picked men for health, and in the prime of life, living under the most favorable conditions for health.

Leicester is a large city, built in a swamp, with the usual variety of conditions of riches and poverty, robust and weakly constitutions, of both sexes and of all ages.

*After* Leicester had discarded vaccination she presents to us this contrast to the army of Bombay :

In the quinquennium 1878–82 there was an annual average of deaths from small-pox of 24 per million ; in that of 1883–87 11 per million, and for the two years 1888–89 *none!* (Table 44, App. 3, *Fourth Report of the Royal British Commission,* page 461.)

There was a great epidemic of small-pox in the city of Bombay in 1872 (a wave of the great pandemic of that period) and vaccination and re-vaccination were pushed. In 1872 about one-fifth of the population had escaped vaccination—" that is to say, until compulsory vaccination was introduced, the residium unvaccinated in Bombay was about one-fifth for the year. The consequence was that in a few years we got an epidemic. Our bad epidemic was in 1872 and after that we got compulsory vaccination and re-vaccination ; *since then we have had no epidemics*" (Q. 1,255).

But in answer to Dr. Collins (Q. 1,340) Dr. Pinkerton says there were 13,404 deaths from small-pox in the Bombay presidency in 1883! And again (Q. 1,415) in 1884 there were 14,438 deaths from small-pox !

I have presumed to say I don't believe Dr. Pinkerton's story about the Hyderabad village, neither evidently did Mr. Picton, Dr. Collins, or Mr. Bradlaugh, members of the Commission, who proceeded to probe it as far as it could be probed. In answer to Mr. Picton we find that this remarkable story was not obtained by census, but "officially" through his vaccinators. (Q. 1,318.) He could not give any numbers. (Q. 1,319.) He did not consider it a very extraordinary occurrence that not one vaccinated child took it. After this Dr. Collins took a hand at the probing. Dr. Pinkerton remembered reporting the case (Q. 1,389 and 1,390). Dr. Pinkerton does not think his report on this case got into print (Q. 1,391). "This would be about as special a case as has come to your notice, would it not?" "YES, THE MOST SPECIAL." Again (Q. 1,392) in answer to Bradlaugh: "And in searching to refresh your memory before coming here you do not find it in any of the official reports?" "No I could not find it."

With cruel irony Mr. Bradlaugh requested him, as he was about to return to India, to furnish the Commission with whatever report on the case he could find.

On page 230, of the *Fourth Report*, Appendix I, that report is furnished. Instead of every unvaccinated Hindu child being attacked, the report gives the number at 19, and nothing whatever is stated as to what occurred among the vaccinated Mussulmans!

Question 1,347 introduces an extract from the thirteenth official report *On Sanitary Measures in India*, 1879–1880, p. 142: "The vaccination returns throughout India show the same fact, that the number of vaccinations does not necessarily bear a ratio to the small-pox deaths. Small-pox in India is related to season and to epidemic prevalence; it is not a disease, therefore, that can be controlled by vaccination in the sense that vaccination is a specific against it. As an endemic and epidemic disease it must be dealt with by sanitary measures, and if these are neglected small-pox is certain to increase during epidemic times." Dr. Pinkerton would not indorse that statement at all.

Question 1,353 introduces an extract from the report of the Army Commission of the Punjaub for 1879, p. 186: "Vaccination in the Punjaub, as elsewhere in India, has no power, apparently, over the course of an epidemic."

Question 1,354 introduces an extract from the report of the central provinces, p. 206 : "The past comparative immunity of the population had been attributed to efficient vaccination, and the people had accepted this protection, but their confidence had been shaken by the reappearance of a severe form of this disease. The Sanitary Commissioner states that he directed a special report on the subject to be made, with the following result: During the early part of the year there had been a good deal of chicken-pox in Sambulpur town; that when small-pox broke out, later on, it attacked those who had been inoculated, vaccinated, and had previously had small-pox or chicken-pox, 301 persons who had been inoculated took the disease; that 577 vaccinated persons were attacked, and 729 unprotected persons, or 1,607 in all." Dr. Pinkerton stated that he knew nothing at all about it, yet he was a Surgeon-General in the service of the Government of India. In response to Q. 1,355 Dr. Pinkerton states that the European army *has more advantages as regards vaccination* than the native army. Q. 1,356 thus introduces an extract from the report of 1883–4, p. 3, and asks if it refers to the European or to the native troops? It says, "There was a greater prevalence of small-pox than usual in the Bengal army. The total cases numbered 105 against 44 in 1882, and 9 deaths against 4. The increase mainly occurred in the Bengal group of stations, where 86 cases were admitted into hospital, the largest number of any year since 1870, and 7 deaths, equal to a ratio of 0.21 per 1,000 of strength." With singular inconsequence Dr. Pinkerton replies, "You might read the sequence to that." "The Madras and Bombay armies recorded one death each." "So far as I know vaccination is not compulsory in the Bengal army. In Bombay it virtually is and has been compulsory for many years; I think probably for thirty years. I do not think that it has ever been compulsory in the Bengal army."

Thereupon Sir James Paget rushes in to help his tottering witness and "to save vaccination from reproach" by asking Q.

1,357 : " And that is the part of the army in which the deaths occurred ?" " Yes, that is so." This paragraph, after giving these details in the Bengal army, finishes by saying : " The Madras and Bombay armies recorded one death each." Cruel Dr. Collins renews his attack. (Q. 1,358.) " Might I repeat the question that I asked ? Do these figures relate to the native or to the European army ?" " They relate to the native army, I think. The Bengal army means the native army, usually." Thereupon Dr. Collins assaults the Commission on the flank, and takes Dr. Pinkerton in the rear. (Q. 1,359.) " If you turn over the page you will see that it is headed 'European Army, India.' " " Yes, it is the European army." I would be glad to leave Dr. Pinkerton here ; who is, I dare say, a highly honorable gentleman in all other respects, but one of the most important lessons to be learned from the entire history of the vaccination superstition is the demoralization which has been effected in the profession through the state support it has received. Dr. Pinkerton, in answer to Q. 1,355, claimed that the European army had had *more* advantages as regards vaccination than the native army, but now (Q. 1,361) he is only certain as to the revaccination of the European army of Bombay ; he *thinks*, but is not certain, that it is the case with the Bengal European army.

Q. 1,377 introduced the following extract from one of the reports on sanitary measures in India up to June, 1886, Vol. XVIII, p. 203, referring to the sanitary measures of the northwest province and Oudh :

" The facts already stated show conclusively that the small-pox of 1884 was one of the most severe epidemics on record, and by *far the most severe* in these provinces since deaths were registered." (Compare answer to Q. 1,255, *supra*, p. 81.) " We are thus brought face to face with the fact, that notwithstanding the existence of an active vaccination service, small-pox swept over the provinces just as if there had been none. * * * If sanitary work be neglected, no more dependence against small-pox epidemics can be placed on vaccination than can be placed on quarantine against invasion of cholera. The true remedies lie elsewhere altogether."

This report was made by Surgeon-General Dr. B. Simpson, Sanitary Commissioner of the Government of India, who yet managed to continue to believe, or to think that he believed, that vaccination is really prophylactic to some degree against small-pox. And Dr. Pinkerton, on his side, persisted that, although sanitation is a good thing generally, he doubts very much whether it has any influence upon small-pox deaths.

It is important, in view of one of the claims set up in support of vaccination, to observe that Dr. Pinkerton states (Q. 1,421) the vast majority of vaccinations to be performed on children over two years, and (Q. 1,422) that he thinks small-pox in India is becoming less an infantile disease than it was.

Here we take leave of Surgeon-General Dr. John Pinkerton. The next witness before the Commission was Dr. Arthur F. Hopkirk, an Englishman, but M. D. of the University of Jena. We shall find him a very interesting witness—as a psychological study.

